# Incidence, Treatment, and Survival Patterns for Sacral Chordoma in the United States, 1974–2011

**DOI:** 10.3389/fonc.2016.00203

**Published:** 2016-09-12

**Authors:** Esther Yu, Paul P. Koffer, Thomas A. DiPetrillo, Timothy J. Kinsella

**Affiliations:** ^1^Radiation Oncology, Tufts Medical Center, Boston, MA, USA; ^2^Radiation Oncology, Warren Alpert Medical School of Brown University, Providence, RI, USA

**Keywords:** chordoma, IMRT, protons, SBRT, heavy ion

## Abstract

**Introduction:**

Sacral chordomas represent one half of all chordomas, a rare neoplasm of notochordal remnants. Current NCCN guidelines recommend surgical resection with or without adjuvant radiotherapy or definitive radiation for unresectable cases. Recent advances in radiation for chordomas include conformal photon and proton beam radiation. We investigated incidence, treatment, and survival outcomes to observe any trends in response to improvements in surgical and radiation techniques over a near 40-year time period.

**Materials and methods:**

Three hundred forty-five microscopically confirmed cases of sacral chordoma were identified between 1974 and 2011 from the surveillance, epidemiology, and end results program of the National Cancer Institute. Cases were divided into three cohorts by calendar year, 1974–1989, 1990–1999, and 2000–2011, as well as into two groups by age ≤65 versus >65 to investigate trends over time and age *via* Chi-square analysis. Kaplan–Meier analyses were performed to determine effects of treatment on survival. Multivariate Cox regression analysis was performed to determine predictors of overall survival (OS).

**Results:**

Five-year OS for the entire cohort was 60.0%. OS correlated significantly with treatment modality, with 44% surviving at 5 years with no treatment, 52% with radiation alone, 82% surgery alone, and 78% surgery and radiation (*p* < 0.001). Age >65 was significantly associated with non-surgical management with radiation alone or no treatment (*p* < 0.001). Relatively, fewer patients received radiation between 2000 and 2011 compared to prior time periods (*p* = 0.03) versus surgery, for which rates which did not vary significantly over time (*p* = 0.55). However, 5-year OS was not significantly different by time period. Age group and treatment modality were predictive for OS on multivariate analysis (*p* < 0.001).

**Conclusion:**

Surgery remains an important component in the treatment of sacral chordomas in current practice. Fewer patients were treated with radiation more recently despite advances in photon and proton beam radiation. OS remains unchanged. Additional analyses of margin status, radiation modality, and local control in current practice are warranted.

## Introduction

Sacral chordomas represent approximately half of all chordomas, a rare neoplasm of the notochordal remnants. They comprise 1–4% of all primary bone tumors (1). Current NCCN guidelines recommend surgical resection with or without adjuvant radiotherapy or definitive radiation for unresectable cases. However, en bloc resections with negative margins can be difficult to achieve and result in significant long-term morbidity with impaired bowel, urinary, and ambulatory function (2). With surgery alone, local control rates range from 35 to 50% but are improved with adjuvant radiation (3–6).

Recent advances in radiation in the past 10–15 years for chordomas include conformal photon, proton, and heavy ion therapy with improving local control rates and functional outcomes (7–9). We investigated the incidence, treatment, and survival outcomes associated with sacral chordomas to elucidate any trends over a near 40-year time period from 1974 to 2011 in response to improvements in surgical and radiation techniques.

## Materials and Methods

Patients with microscopically confirmed chordoma confined to the pelvis and sacrum were identified in the surveillance, epidemiology, and end results (SEER) database from 1974 to 2011 by selecting all cases using the ICD-03 code 9370/3: Chordoma, NOS. We further specified disease site with the following codes localizing to pelvis, coccyx, and, sacrum: C41.4 and C49.5. Incidence and survival rates were adjusted for age. Cases were divided into three cohorts by calendar year, 1974–1989, 1990–1999, and 2000–2011, as well as into two groups by age ≤65 versus >65 to investigate treatment characteristics by time and age *via* chi-square analysis. Univariate chi-square analyses were performed to determine predictors of the use of surgery or radiation. Kaplan–Meier analyses were performed and compared *via* log-rank test to determine the effects of treatment on overall survival (OS). Multivariate Cox regression analysis was performed to determine predictors of OS. All statistical analyses were performed in SPSS version 20.

## Results

Three hundred forty-five microscopically confirmed cases of sacral chordoma were identified between 1974 and 2011 from the SEER program of the National Cancer Institute. The age-adjusted incidence rate of sacral chordomas was 0.03 per 100,000. The 5-year relative survival for the entire cohort was 60.0%. Median age at diagnosis was 64 years. Treatment characteristics of the entire cohort are listed in Table 1. Overall, 67% of patients had surgery with or without radiation, and a minority of patients were treated with radiation alone.

**Table 1:** **Treatment characteristics**.

Treatment modality	Number (%)
No treatment	43 (13)
Surgery alone	150 (43)
Surgery and radiation	83 (24)
Radiation alone	47 (14)
Unknown	22 (6)

On univariate analyses for factors associated with use of radiation, only time period correlated significantly with the use of radiation, with decreasing utilization over time (Table 2). For surgery, only age ≤65 correlated with the receipt of surgery (Table 3).

**Table 2:** **Factors associated with radiation**.

Univariate analyses	*p*-Value
Age	0.5
Sex	0.4
Race	0.8
Tumor size	0.07
Time period (1974–1989, 1990–1999, and 2000–2011)	0.03

**Table 3:** **Factors associated with surgery**.

Univariate analyses	*p*-Value
Age	<0.01
Sex	0.3
Race	0.3
Tumor size	0.9
Time period (1974–1989, 1990–1999, and 2000–2011)	0.5

**Figure 1:**
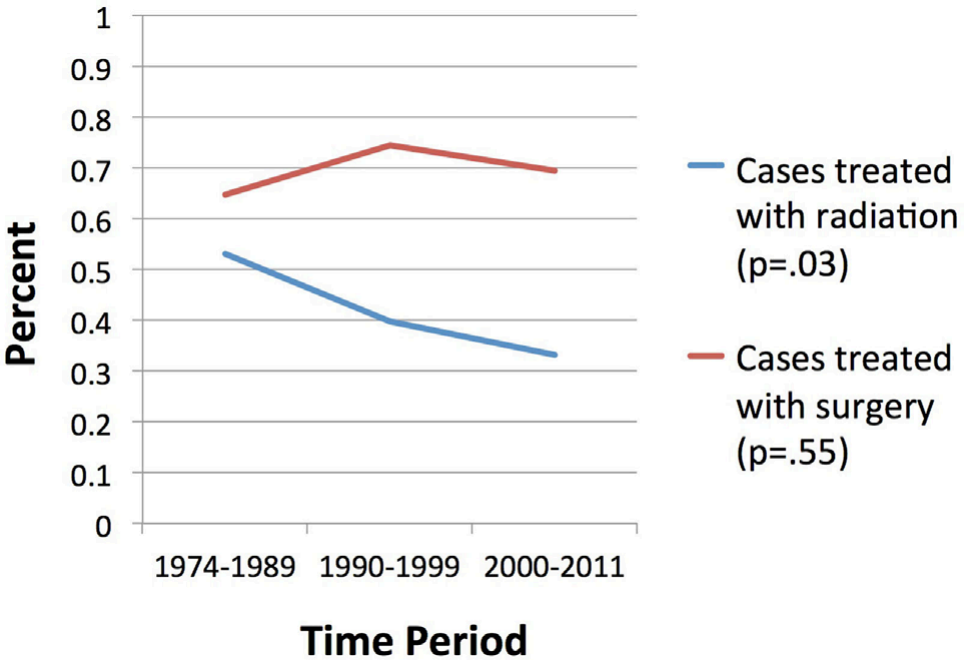
**Trends in surgery and radiotherapy use**.

**Figure 2:**
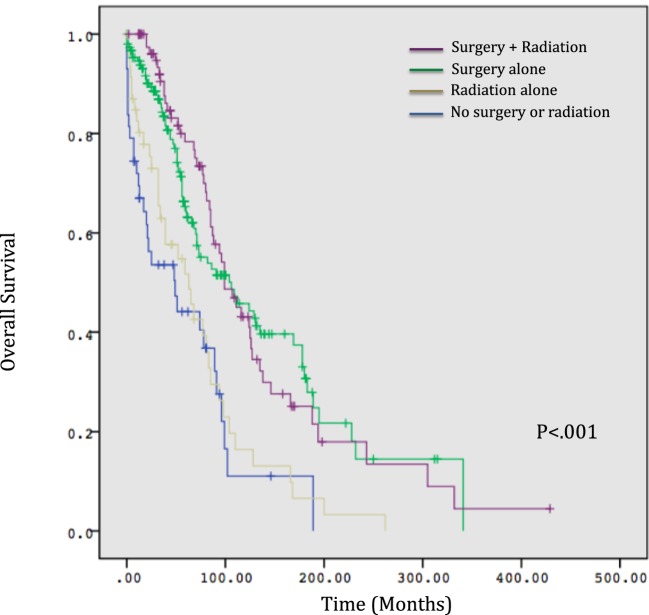
**Overall survival by treatment modality**.

Overall survival correlated significantly with treatment modality, with patients treated with surgery alone with or without radiotherapy experiencing longer estimated median survivals compared to those receiving radiotherapy alone or no treatment (104 and 99 months versus 63 and 49 months, respectively, *p* < 0.001, Figure 1). Age >65 did not correlate with any difference in OS (*p* = 0.63) but was significantly associated with non-surgical management with radiotherapy alone or no treatment (*p* < 0.001). A significantly smaller proportion of patients received radiotherapy between 2000 and 2011 compared to prior time periods (*p* = 0.03) versus surgery, for which rates which did not vary significantly over time (*p* = 0.55) (Figure 2). OS was 63% for patients diagnosed from 1974 to 1989, 59% from 1990 to 1999, and 63% from 2000 to 2011 (*p* = 0.50).

On multivariate analysis, age group and treatment modality were significant predictors of OS (*p* < 0.001). Surgery alone and the combination of surgery with radiation were predictive of OS with a hazard ratio of 0.46 and 0.47, respectively (*p* < 0.001, 95% CI 0.30–0.72 for surgery; *p* < 0.001, 95% CI 0.29–0.77 for surgery and radiation). Sex and time period were not predictive for OS (*p* = 0.36 and *p* = 0.52, respectively).

## Discussion

Chordomas are rare tumors occurring less than one per one million people. Our observed age-adjusted rates were in keeping with those reported in the literature. The standard of care in the treatment of sacral chordoma is an en bloc sacrectomy with negative margins. Gross total resection can result in high rates of neurologic deficits with significant long-term morbidity with urinary and fecal incontinence and pain (1), and patients remain at high risk of local failure even with wide margins (3, 10–12). As such, radiation therapy is often a crucial component of adjuvant treatment of resected tumors or as primary treatment for medically inoperable tumors.

Radiation technology has undergone numerous advancements in the past 10–15 years, allowing for the treatment of tumors while minimizing normal tissue toxicity. Various modalities that are now used include intensity-modulated radiation therapy (IMRT), proton beam radiation, heavy ion radiotherapy, and stereotactic body radiation therapy (SBRT) (7, 13–16). As such, the purpose of this study was to determine if the patterns of care as reported in the SEER database have changed to reflect the increasing number of radiation options in the primary or adjuvant setting. Over the past 35 years, we found that the use of surgery has not changed over time, with 67% of patients undergoing resection that did not vary significantly over the three time periods. However, we found that the use of radiation has surprisingly decreased over time. Despite this finding, we found that OS remained unchanged over time. The only predictor in univariate analysis for the utilization for radiation was time period.

The reason for the decreasing utilization of radiation is unclear. Information on margin status is unavailable in the SEER database, and thus improvement in surgical and imaging technology potentially allowing for greater resection with negative margins is one possible explanation. Whether recent advances in imaging technology corresponded any differences over time in tumor size at diagnosis could not be determined as tumor size was only reported in the SEER database beginning 2004. It is possible that rates of utilization of particular radiation modalities such are changing over time, but utilization of specific radiation modalities, whether IMRT, or proton beam, or SBRT, is unable to be determined from the SEER database.

Despite the decreasing trend toward radiation therapy, OS remains unchanged over time, both on univariate and on multivariate analysis. This may relate to the relatively constant rates of surgical resection. However, whether local control, which is often difficult to achieve with surgery alone, also remains constant over time cannot be determined from the SEER database. Local control may correlate with OS in patients who eventually succumb to uncontrolled primary disease (2), but patients often present at an older age with significant comorbid illnesses that can compete for survival (2). This is consistent with our univariate analysis in which age predicted for treatment with surgery with or without radiation and was predictive of OS on multivariate analysis. Performance status can also dictate treatment modality and especially the choice for surgery in clinical practice and may independently predict for OS, but performance status is not reported SEER database. OS may not necessarily reflect the consequences of changing practices in radiation, and the lack of change in OS may simply represent the relatively unchanged competing causes of death in this elderly population. The median age for diagnosis in the examined cohort was 64 years, but no medical comorbidity data and cause-of-death information are reported in the SEER database to determine disease-specific survival.

Chordomas have historically been described as radioresistant, requiring at least 60 Gy with standard, fractionated external beam radiation to achieve durable local control (17). With higher doses of radiation, toxicities of treatment, especially gastrointestinal and urinary, can be considerable in patients who may already have diminished function secondary to their tumor and/or surgery. However, the development of newer techniques, such as intensity modulation and stereotactic radiation, along with proton beam radiation may allow for dose escalation to maximize local control while minimizing dose to regional bowel and bladder. Zabel-du Bois et al. report a 1-year local control rate of 79% in 17 sacral chordoma patients treated postoperatively and definitively with IMRT with a median dose of 54 Gy with significantly improved local control in patients treated >60 Gy. However, at 5 years, local control was 27% with OS 70% (9) in keeping with historical rates of local control with conventional radiation techniques.

Proton beam radiation and heavy ion therapy afford sharp dose fall-off owing to the Bragg peak phenomenon in which protons deposit dose at the end of their range in tissue, allowing dose escalation to the tumor while minimizing dose to organs at risk. DeLaney et al. reported on 59 patients with spine chordomas, chondrosarcomas, and other sarcomas (including 58% chordomas), treated with surgery and/or photon/proton beam radiation to a median relative biological effectiveness (RBE) dose 76.6 Gy. With median follow-up of 7.3 years, 8-year local control was 85% for primary tumors of the spine, with 13% late grade 3–4 toxicity (7). Park et al. examined a cohort of 27 primary and recurrent sacral chordomas also treated with photon/proton radiation with or without resection. Primary tumors were treated with a mean dose of 71 and 77 Gy for recurrent chordoma. At 5 and 10 years, local control was 72 and 57.5% and OS 82.8 and 62.5%, respectively (18). Despite these improvements in local control with proton beam radiation, the benefits may yet be offset if the adjuvant radiation is not delivered in a timely fashion. In 19 chordoma patients in which negative margins could not be achieved with resection, 5-year local control with proton beam radiation to median 70 GyRBE was 9% in patients undergoing salvage radiation, as opposed to 88% with early adjuvant radiation. While 2-year OS was not statistically significantly different, this study nonetheless highlights the importance of timely adjuvant intervention to achieve local control in high-risk patients (16).

Heavy ions, including carbon ions, have a higher linear energy transfer (LET) than both photons and protons and can potentially have greater radiobiological effect on tumor. Single institutions worldwide have employed carbon ions with excellent outcomes in sacral chordoma. Imai et al. saw a 5-year local control rate of 77% in 188 patients with unresectable chordomas treated with doses ranging from 64 Gray-equivalent (GyE) to 73.6 GyE. On univariate analysis, there was no difference in local control with dose >70.4 GyE, as compared to <67.2 GyE. Nishida et al. retrospectively analyzed 17 patients treated with surgery or carbon ion radiotherapy to a median dose 70.4 Gy (19). With 76-month median follow-up, the authors reported local recurrence-free survival 62.5% for patients treated with surgery versus 100% with radiation. Anorectal and urinary function declined in 60% of patients undergoing surgery but remained unchanged in all patients treated with radiation. While these outcomes are subjected to selection bias, the authors report no differences in both groups with regard to the number of tumors ≥10 versus <10 cm and also frequency of tumor extension to the L5–S1 or S2–5 nerve roots (14).

Stereotactic body radiation therapy is a treatment modality in which high doses of radiation are delivered (typically in one to five fractions) to a target within the body with a high level of precision (20). SBRT has been used in treating skull base chordomas with good outcomes (21) and is under investigation in treating sacral chordomas. Henderson et al. reported 5-year 59.1% local control in 18 chordoma patients, 3 with sacral disease with Cyberknife as adjuvant or primary therapy. Patients were treated with five fractions with fractional doses ranging from 7 to 8 Gy per fraction. No grade 3 or 4 complications were reported, and mean pain scores were improved even in patients with long-term follow-up. The authors estimate the α/β ratio of chordoma to be approximately 2.45, thereby predicting improved outcomes with hypofractionation (22). A retrospective analysis of 24 patients, 10 of whose tumors were located in the sacrum or pelvis, treated with a single fraction of stereotactic radiation revealed a radiographic local control rate of 95% at 2 years. Patients received a median dose of 24 Gy as part of preoperative, definitive, or adjuvant treatment. One patient with sacral disease with known sciatic nerve invasion developed late sciatic neuropathy with foot drop, while two developed sacral fractures. The authors conclude that single-fraction SBRT may safely treat chordoma and may be a treatment options for patients unsuitable to wide margin excision or as preoperative treatment (13).

Given the availability of more radiation options that afford higher rates of local control as compared to conventional photon irradiation in the past, there may potentially be differing rates of utilization of various modalities, whether SBRT or proton beam radiation, but this has yet to be shown as we are unable to determine radiation modality from the SEER database. Furthermore, whether local control rates have changed in light of the improved radiation techniques despite decreasing utilization also remains unknown. Correspondingly, whether patients treated definitively in the recent decade have benefited from the improved technology from a functional standpoint, and quality of life remains to be seen. However, we conclude that surgery remains an essential component in the treatment of sacral chordomas. Fewer patients appear to be undergoing radiation therapy with or without surgery more recently, but OS remains unchanged over time.

## Author Contributions

EY – hypothesis generation, statistical analysis, manuscript preparation, and editing. PK – manuscript editing and statistical analysis. TK – manuscript editing and advising. TD – manuscript editing and advising.

## Conflict of Interest Statement

The authors declare that the research was conducted in the absence of any commercial or financial relationships that could be construed as a potential conflict of interest.
